# Supplementary material for: Learning curve and surgical outcome of robotic assisted colorectal surgery with ERAS program
Source: Sci Rep. 2022 Nov 29;12:20566. doi: 10.1038/s41598-022-24665-w (PMC9709162; doi:10.1038/s41598-022-24665-w)
Supplement: Supplementary file 1 — Supplementary Information 1. [file 41598_2022_24665_MOESM1_ESM.zip › LSC Raw data-Ñ[▒K/114/H_ñw╝╨░O▒Kñσ.pdf]

正式摘要(電子簽章：000000210066646)

W52 / W52 ☐ 英文標題(注意:無英文姓名-請補病人基本檔!) ☐【讀取】憑證

臺中榮民總醫院

出院病歷摘要

轉入號 : 轉出號 :

[redacted]

(8)流水編號	
(9)入院日期	110/01/20 (2021) 直腸外科 W52 - 122
(10)轉科日期	
(11)出院日期	110/02/03 (2021) 住院天數計 14 日

診斷

- 入院 : 1.S-colon tumor with obstruction
  2.hypertension
  3.hyperlipidemia
  4.type 2 diabete mellitus
- 出院 : 1.S-colon tumor with obstruction s/p anterior resection on 2021/1/21 with wound infection s/p wet dressing
  2.hypertension
  3.hyperlipidemia
  4.type 2 diabete mellitus

主訴

- abdominal fullness for about 2 months

病史

- This 77 years old female patient has past history of hypertension, hyperlipidemia, type 2 diabete mellitus and ATH for uterine myoma 40 years ago. She complained abdominal fullness and body weight loss since Nov. 2020. She came to 部立苗栗醫院 on Dec. 2020 for help initially. Elevated CEA(17.3) was noted. Colonoscopy showed sigmoid colon tumor 40 cm from anal verge. Adenocarcinoma was proved in biopsy. Abdmonial CT showed tumor sized about 7 cm with regional lymphadenopathy without distal metastasis. Thus, she was transferred to our hospital for surgical intervention.

體檢發現

-
  - General appearance :
    - easy looking , conscious clear , without cardiopulmonary distress
    - Vital signs自動帶入date 體溫(BT) 脈搏(P) 呼吸(R) 血壓(BP) 身高(BH) 體重(BW) BMI
      20210120 36℃ 102次/分 18次/分 134/66mmHg 151cm 47kg 20.6
  - HEENT :
    - Eyes: no anemic conjunctiva, no icteric sclera, normal EOM
    - ENT: no otorrhea, no rhinorrhea, no erythematous mucosa, no injected tonsil, no deformity
  - Neck: supple, freely movable, trachea midline,no deformity, no engorged jugular vein , no palpable thyroid or lymph node enlargement
  - Chest:
    - Inspection: Grossly normal, symmetric and fully expansion, no accessory muscle used, no operation scar, no spider angiomata, no gynecomastia
    - Palpation: no palpable mass or nodule, no subcutaneous emphysema, no tenderness
    - Ausculation: clear breathing sound
  - Heart:
    - Percussion: normal heart size
    - Ausculation: regular heart beat, normal S1, S2, no gallop, no murmur
  - Abdomen:

-
  - Inspection: in
    - flat contour, symetrically, no scar, no stria, no dilated veins, no rashes
  - Ausculation: normoactive bowel sound, no abdominal bruits
  - palpation: soft, no muscle guarding or rigidity, no local tenderness, no rebounding pain, no palpable mass, no palpable pulsation
  - Percussion: no shifting dulllness, no hepatomegaly, no splenomagaly

-

Peripheral pulsation :	CA	BA	RA	FA	PA	PTA	DPA
Right side :	++	++	++	++	++	++	++/+++
Left side :	++	++	++	++	++	++	++/+++

- Genito-anus: no tenderness or palpable mass lesion or rectal shelf, normal external genitalia, normal anal tone, brown stool

CRS CONDITION

- Colonoscope report: sigmoid colon tumor 40 cm from anal verge
- CEA: 17.3 ng/ml
-
  - Lung

手術日期及方法

- 手術日期及方法20210121 1. Conventional, left anterior resection. (Initially tried laparoscopic)
  2. Intraoperative colonoscopy : (-)
  3. IMA high ligation (+). Left colic vessels were also ligated. (Middle colic vessels were not approached.)
  4. EndoGIA (60-4.1) X1 + EEA (33mm) were applied for end to end anastmosis
  5. The colon was taken down from the splenic flexure site and resected
  6. Proximal cut end : > 10 cm, distal cut end : 7 cm
  7. One drainage tube (12 Fr. penrose) was placed over the pelvic (Anastomosis site) area from the LLQ of abdomen
  8. ADHESION BARRIER FILM was placed below the central abdominal wound.

住院治療經過

- After admission, operation with anterior resection was done on 1/21. We let her try feeding as her tolerance. We let her try water and then diet as her tolerance. IVF was stopped under well oral intake. Penrose drain was removed on POD5. Due to abdominal wound discharge, we kept wet dressing since 1/29, on 2/2 the wound condition was well, under stable condition, the patient was discharge with OPD follow up.

合併症

一般檢查

- 累積報告 :

DATE	TIME	ALB	TP	BIL.T	BIL.D	ALKP	AST	ALT	LDH	CHOL	TG	HDL-C	LDL	UA	NH3	AMY
1100120	2140		6.6							217	101	44	152	4.5		
1100121	2047	2.5		0.69			16	9								
1100128	0846	2.6		0.34				6								

DATE	TIME	WBC	RBC	HGB	HCT	MCV	PLT	BLST	NEUT	BAND	SEG	LYM	MON	EOS	BAS
1100121	2046	9780		10.3	32.4		247		87.3			10.7	1.8	0.0	0.2
1100125	0933	10060		10.8	34.6		245		68.5			23.2	5.1	2.7	0.5
1100128	0934	7670	4.12	10.3			257		64.8			23.5	6.5	4.8	0.4
1100201	1031	7110		10.1	31.7		298		65.1			23.3	6.0	4.9	0.7

DATE	TIME	NA	K	CL	CA	BUN	CREAT	Mg	P	CRP	ESR	PCT	LIPASE	LACTATE
1100120	2140									0.813				
1100121	2047	137	3.0		7.9	9	0.61							
1100125	1021	141	3.6			8	0.60							
1100128	0846	141	3.3	103	8.1		0.59							
1100201	0924	141	3.3			8	0.55							

特殊檢查

病理報告

其它

出院時情況

- 3.改門診治療

出院指示

-

藥名	劑量	劑量單位	用法	頻次	數量	數量單位	現況	天數	單位
Ulstop tab 20mg	1	TAB	PO	BID	14	TAB	TH	7	天
20mg Lipitor tab	1	TAB	PO	QD	7	TAB	TH	7	天
10/160/25mg Exforge HCT	1	TAB	PO	QD	7	TAB	TH	7	天
Acetylcysteine tab	1	TAB	PO	QN	7	TAB	TH	7	天
Voren-K tab 25mg	1	TAB	PO	TID	21	TAB	TH	7	天
5mg Bisoprolol tab	1	TAB	PO	QD	7	TAB	TH	7	天
Amaryl tab 2mg	1	TAB	PO	QDAC	7	TAB	TH	7	天
Mag. Oxide tab	2	TAB	PO	TID	42	TAB	TH	7	天

出院計劃

主治醫師 2062F 蔣鋒帆 / 住院醫師 2332G 林志安

正式摘要(電子簽章：000000210066646)

W52 / W52

doctorRank:B
